# Cytogenomic assessment of the diagnosis of 93 patients with developmental delay and multiple congenital abnormalities: The Brazilian experience

**DOI:** 10.6061/clinics/2017(09)02

**Published:** 2017-09

**Authors:** Évelin Aline Zanardo, Roberta Lelis Dutra, Flavia Balbo Piazzon, Alexandre Torchio Dias, Gil Monteiro Novo-Filho, Amom Mendes Nascimento, Marília Moreira Montenegro, Jullian Gabriel Damasceno, Fabrícia Andreia Rosa Madia, Thaís Virgínia Moura Machado da Costa, Maria Isabel Melaragno, Chong Ae Kim, Leslie Domenici Kulikowski

**Affiliations:** I Laboratorio de Citogenomica, Departamento de Patologia, Faculdade de Medicina FMUSP, Universidade de Sao Paulo, Sao Paulo, SP, BR; II Departamento de Morfologia e Genetica, Universidade Federal de Sao Paulo, Sao Paulo, SP, BR; III Unidade de Genetica, Departamento de Pediatria, Instituto da Crianca, Hospital das Clinicas HCFMUSP, Faculdade de Medicina, Universidade de Sao Paulo, Sao Paulo, SP, BR

**Keywords:** Cytogenomic Techniques, MLPA, Array, Developmental Delay, Multiple Congenital Abnormalities

## Abstract

**OBJECTIVE:**

The human genome contains several types of variations, such as copy number variations, that can generate specific clinical abnormalities. Different techniques are used to detect these changes, and obtaining an unequivocal diagnosis is important to understand the physiopathology of the diseases. The objective of this study was to assess the diagnostic capacity of multiplex ligation-dependent probe amplification and array techniques for etiologic diagnosis of syndromic patients.

**METHODS:**

We analyzed 93 patients with developmental delay and multiple congenital abnormalities using multiplex ligation-dependent probe amplifications and arrays.

**RESULTS:**

Multiplex ligation-dependent probe amplification using different kits revealed several changes in approximately 33.3% of patients. The use of arrays with different platforms showed an approximately 53.75% detection rate for at least one pathogenic change and a 46.25% detection rate for patients with benign changes. A concomitant assessment of the two techniques showed an approximately 97.8% rate of concordance, although the results were not the same in all cases. In contrast with the array results, the MLPA technique detected ∼70.6% of pathogenic changes.

**CONCLUSION:**

The obtained results corroborated data reported in the literature, but the overall detection rate was higher than the rates previously reported, due in part to the criteria used to select patients. Although arrays are the most efficient tool for diagnosis, they are not always suitable as a first-line diagnostic approach because of their high cost for large-scale use in developing countries. Thus, clinical and laboratory interactions with skilled technicians are required to target patients for the most effective and beneficial molecular diagnosis.

## INTRODUCTION

The human genome contains several types of structural variations that contribute to genetic diversity and disease susceptibility 1,2. These structural variations include single nucleotide alterations, such as point mutations or SNPs (single nucleotide polymorphisms), small InDels, and copy number variations (CNVs) 1,3.

CNVs are the most prevalent type of structural variation in the human genome and can affect the transcription rate, sequence, structure, and function of genes. These genomic variations include a range of deletions and duplications larger than 1 kb and up to several Mb 1,2.

Although these variations often represent only small genomic segments, they can generate several specific clinical abnormalities, such as developmental delay (DD) and multiple congenital abnormalities (MCAs) 1-4. However, the etiology of these disorders is not well understood, making genetic counseling and treatment difficult 1,2,5.

Different cytogenomic techniques have been used to detect these changes, including the MLPA (multiplex ligation-dependent probe amplification) and array techniques 1,6,7.

MLPA is a technique that is used to detect deletions and duplications in genetic diseases of interest, such as the most common microdeletion/microduplication syndromes and subtelomeric regions 8,9.

This method is considered a faster alternative and is more economically viable than other molecular techniques 3,10, and it allows quantitative genomic screening of target-specific sequences through simultaneous hybridization and amplification via polymerase chain reaction (PCR) using more than 50 different probes in a single reaction 3,8,11,12.

The screening of specific submicroscopic changes via MLPA detects abnormalities in 5 to 10% of patients with a normal conventional karyotype 13-15. Thus, in a single test, the MLPA evaluates patients with characteristics of microdeletion/microduplication syndromes and/or patients with suspected subtelomeric abnormalities 9,15-18.

Although MLPA allows the evaluation of multiple different genomic regions, the main limitation of this technique is the need for a clinical hypothesis to direct the selection of a specific kit for analysis 3,8. In contrast, the array technique does not require a specific clinical diagnosis before use.

The array technique permits the assessment of the CNVs present in the whole genome of a patient in a single reaction with a high level of resolution (∼0.7 kb), depending on the platform, types of probes and how they are distributed in the genome, thus increasing the detection rate of complex imbalances 4,19,20.

This technique involves the hybridization of probes to complementary DNA (genomic sequence segments) on a slide or chip array and subsequent analysis of the fluorescence annealed to the target DNA sequences using specific software 7,21.

Currently, there are several companies that offer this technology on different platforms, offering slides or chips with a high density or coverage of the genome. However, these platforms vary in the number of probes used, and several of them can interrogate millions of regions in a single sample 4,7,20,22,23.

The main advantage of the array technique is the ability to investigate the entire genome in a single experiment with higher resolution and accuracy compared with traditional and molecular cytogenetics, as this allows the investigation of small changes that may have an impact on the phenotype of patients without a definitive clinical diagnosis 19,22,24.

Thus, arrays have been employed to diagnose patients with DD and MCAs as well as normal karyotypes, increasing the detection rate of small genomic imbalances and the diagnosis of patients with clinical phenotypes of unknown etiology 22,25.

The main limitations of the array technique are the high cost of large-scale application for developing countries, the experimental time required (3-5 days), and the expertise required for classification of the results (CNVs), which can only be interpreted by a highly qualified professional 25-27.

An unequivocal diagnosis is fundamental to providing suitable answers regarding the prognosis and risk of recurrence and can contribute to improving public health policy 2,25,28.

In developed countries, the array technique is already being used as the first-line molecular diagnostic test in patients with MCA 28,29. Recently, Brazil has modified its policies in the field of genetics, including the clinical genetics policy guidelines of the *Sistema Único de Saúde* (SUS), and has provided financial incentives to cover the costs of genetic testing and counseling in the national health network (http://bvsms.saude.gov.br/bvs/publicacoes/diretrizes_atencao_integral_pessoa_doencas_raras_SUS.pdf).

Thus, genetic services must study the best strategies for molecular assessment to diagnose each patient referred with DD and MCA, as the introduction of a single molecular diagnostic method, such as array technology, as a first-line assessment method for patients with DD and MCA is impractical in Brazil due to insufficient public investment in the health care system and because low-income patients cannot afford such tests.

In this study, we report our experience with the implementation and assessment of MLPA using different kits, array platforms (Affymetrix, Agilent and Illumina), and probe densities for the molecular diagnostic and scientific analysis of 93 Brazilian patients with DD and MCA.

## MATERIALS AND METHODS

This study involved 93 patients who were evaluated using MLPA and array techniques. The patients presented with DD and MCAs, such as minor facial anomalies, including a high forehead, frontal bossing, broad nasal bridge, low-set ears, ocular hypertelorism, and abnormalities of the eyes, as well as major congenital defects, such as skeletal and genital malformations, heart defects, and structural brain abnormalities.

All patients were previously assessed through conventional cytogenetic analysis to identify their numerical and structural chromosomal abnormalities; metaphase chromosomes were obtained from peripheral blood lymphocyte samples the patients, and G-banding analysis was performed using standard procedures. In each case, twenty metaphase chromosomes were analyzed at a 550-chromosome band resolution (≥5 Mb) and then classified according to the International System for Human Cytogenetic Nomenclature 2013 (ISCN) guidelines.

Genomic DNA was isolated from 3 mL of peripheral whole blood from patients using a commercially available DNA isolation kit (QIAamp DNA Blood Mini Kit^®^, Qiagen, Hilden, Germany) according to the manufacturer’s instructions. The quality and quantity of the DNA samples were determined using a Qubit^®^ 2.0 Fluorometer (Invitrogen, Carlsbad, California, USA), and the integrity of the DNA was ascertained via agarose gel electrophoresis analysis.

All of the genomic DNAs were screened with the following three MLPA kits: for the most common microdeletion/microduplication syndromes, the SALSA MLPA probemix P064-B2 Mental Retardation-1 kit was employed, which includes probes for the 1p36 deletion, Williams-Beuren, Smith-Magenis, Miller-Dieker, 22q11.2 deletion, Prader-Willi/Angelman, Alagille, Saethre-Chotzen, and Sotos syndromes; for subtelomeric imbalances, the SALSA MLPA probemix P036-E1 Human Telomere-3 and SALSA MLPA probemix P070-B2 Human Telomere-5 kits were used, which include subtelomeric probes for all chromosomes (MRC-Holland, Amsterdam, Netherlands).

In several cases, the patients’ genomic DNA samples were also assessed using specific MLPA kits to confirm the observed changes. The kits used in these cases were the SALSA MLPA probemix P250-B1 DiGeorge and SALSA MLPA probemix P356-A1 Chromosome 22q kits, which are specific for chromosome 22, and the SALSA MLPA probemix P029-A1 Williams-Beuren Syndrome kit, which is specific for changes in chromosome 7q11 (MRC-Holland, Amsterdam, Netherlands).

DNA denaturation, hybridization of probes, ligation, and PCR were performed according to the manufacturer’s instructions, as described by Schouten et al. 11. Separation of the amplification products via electrophoresis was performed using an ABI 3500 Genetic Analyzer (Thermo Fisher Scientific, Waltham, Massachusetts, USA), and the data were analyzed using GeneMarker software, version 1.6 (www.softgenetics.com-Softgenetics, State College, Pennsylvania, USA).

The peak area of each fragment was compared with that of a control sample, and the results were considered abnormal when the relative peak-height ratio was less than 0.75 (deletion) or greater than 1.25 (duplication). The details of the regions and probes detected by each kit can be found at www.mlpa.com.

The arrays were employed on three different platforms, from Agilent Technologies (Santa Clara, California, USA), Affymetrix (Santa Clara, California, USA) and Illumina (San Diego, California, USA), which differ in the technology used.

On the Agilent platform, we used the Human Genome CGH Microarray 2x105K slide, containing 105,750 probes with an average spacing of 22 kb, the SurePrint G3 Human CGH Microarray 4x180K slide, containing 180,880 probes distributed throughout the genome with an average spacing of 13 kb, and the SurePrint G3 Human CGH Microarray 8x60K slide, containing 62,976 probes with an average spacing of 41 kb.

On the Affymetrix platform, we used the Affymetrix Genome-Wide Human SNP Array 6.0 chip (1.8 million genetic markers), which contains 906,600 single-nucleotide polymorphism (SNP) probes and over 946,000 probes for the detection of CNVs, with a median physical inter-marker distance of 1-5 kb, as well as the CytoScan HD chip, which contains 2,696,550 CNV probes and 749,157 SNP probes, with an average spacing of 1.1 kb.

On the Illumina platform, we employed the HumanCytoSNP-12 BeadChip, with 300,000 oligonucleotide probes and an average spacing of 9.7 kb, and the CytoSNP-850K, with 843,888 markers and an average probe spacing of 1.8 kb across the whole array.

In all samples, amplification, hybridization, staining and washing were performed according to the manufacturers’ protocols, and the data were extracted by a specific scanner. The CGH arrays are based on the principle of comparison between the signal intensities of a sample and commercially acquired human male control DNA (Promega Corporation, Madison, Wisconsin, USA). For the SNP arrays (Affymetrix) and bead arrays (Illumina), only a single hybridization is performed for the patient DNA, and the signal intensities are then compared with a reference dataset based on pre-run reference samples.

The raw data were analyzed using Feature Extraction v9.5, Affymetrix Chromosome Analysis Suite (ChAS) v.1.2, or KaryoStudio v1.4.3.0 Build 37 software. The data were normalized, and log_2_ ratios were calculated by dividing the normalized intensity of the sample by the mean intensity across the reference sample.

The criteria used to determine a CNV included the involvement of at least five consecutive probes sets in a region and log2 ratio cut-offs of -0.41 and +0.32 for loss and gain, respectively. The software produced graphical representations of CNV breakpoints for each sample.

The SNP and bead arrays supply the B allele frequency (BAF), which represents the proportion of B alleles in the genotype. A region without evidence of CNVs should show a log_2_ ratio near zero and three BAF clusters of 0, 0.5, and 1, corresponding to the AA, AB, and BB genotypes, respectively.

All samples were evaluated and were found to be in accordance with the quality standards.

The results were analyzed according to the American College of Medical Genetics guidelines 30 using independent tests and were compared with the following databanks of CNVs and classified as benign, pathogenic or VOUS (variants of uncertain clinical significance): the Database of Genomic Variants (DGV – http://projects.tcag.ca/variation/), the Database of Chromosomal Imbalance and Phenotype in Humans Using Ensembl Resources (DECIPHER – http://decipher.sanger.ac.uk/) and the UCSC Genome Bioinformatics database (http://genome.ucsc.edu). The genomic positions are reported according to their mapping on the GRCh37/hg19 genome build.

### Ethics

The Research Ethics Committee of the Hospital das Clínicas da Faculdade de Medicina da Universidade de São Paulo (HC-FMUSP) approved this study, and written informed consent for publication was obtained from the parents of the patients (CAPPesq n° 0619/11).

## RESULTS

In this study, we assessed 93 patients with DD and MCAs via the MLPA and array techniques. The patients showed either a normal karyotype or a karyotype with an undetermined abnormality according to G-banding, which made it impossible to obtain a conclusive diagnosis.

**Figure 1:**
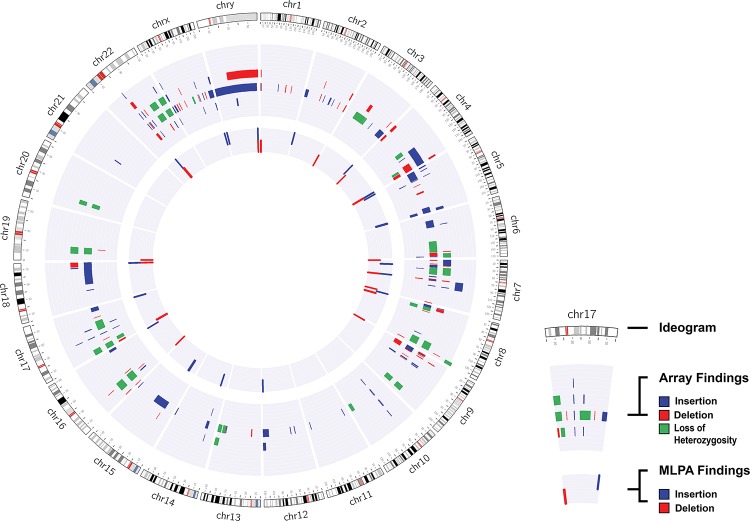
Cytogenomic map of the raw data of all alterations identified via the MLPA and array techniques. The gray circles represent the locations of the breakpoints of the alterations identified by both techniques, in which the center circle corresponds to the MLPA results and the middle circle to the array results. Each bar refers to the position of each identified copy number change: the red bar refers to deletions, the blue to duplications, and the green to loss of heterozygosity. The genomic positions are reported according to their mapping on the GRCh38/hg38 genome build from the UCSC Genome Browser.

We found that ∼97.8% (91/93) of the results from the two methods were consistent with each other (all results are described in Table 1). Among the evaluated patients, ∼13.2% (12/91) showed no alterations according to either technique; ∼54.9% (50/91) only showed changes in the array analysis; and ∼39.9% (29/91) of the patients showed CNVs according to both techniques (Figure 1).

One case with inconclusive results was found in our cohort, and further evaluation using other molecular techniques should be performed to definitively diagnose this patient. Although the changes observed using both techniques were consistent, the breakpoint determined by the array did not correspond exactly to the genomic localization of the MLPA probe, and there were several array probes between these two probes.

The MLPA results were inconsistent with the array results in two cases. We found a duplication in the *FZD9* gene in one case (P064 and P029), and in the other, we identified two alterations (del 16p13.3 with the P036 kit and del 19p13.3 with the P070 kit) using MLPA, which were confirmed via independent reactions. However, these alterations were not identified with the array because none of the array probes are located at exactly the same position as the MLPA probe.

Several of the MLPA results were inconclusive, but this did not affect the comparison of the techniques because the regions targeted by MLPA were repeated in several of the kits used in this study. Thus, the results were concordant, and although the results were not the same in all cases, the MLPA technique detected ∼70.6% of the pathogenic CNVs detected using the array.

### MLPA Analysis

The MLPA technique was employed to diagnose all patients using several different kits. No changes were detected in ∼66.7% (62/93) of the patients, and in four cases, one or two kits showed inconclusive results; however, these cases did not influence the assessment and interpretation of the results.

**Figure 2:**
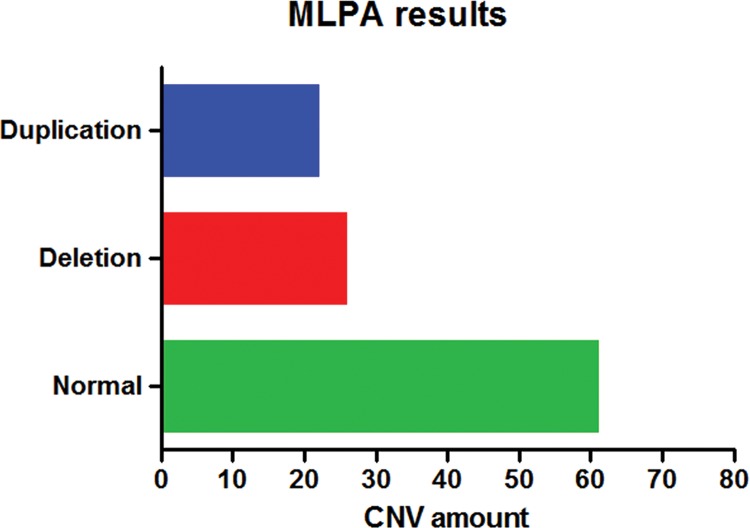
The results of MLPA. The blue bar indicates the number of duplications; the red bar indicates deletions; and the green bar indicates the number of normal results detected via MLPA.

CNVs were detected with at least one of the kits in ∼33.3% (31/93) of patients (Figure 2). Approximately 22.6% (7/31) of these changes were detected by the P064 kit, corresponding to one deletion typical of the Williams-Beuren syndrome, one duplication in chromosome 7q11, and five deletions of 22q11.2, which were atypical in three patients and typical in the other two patients. All alterations were confirmed by the specific P029, P250 and/or P356 kits.

We also detected subtelomeric alterations in ∼45.2% (14/31) of the patients. One deletion was detected in two patients; two duplications in different chromosomes were detected in one patient; two deletions were found in another patient, one of which was detected with the P036 kit and the other with the P070 kit; and the remaining 10 patients showed concomitant deletions and duplications, all of which were present in the subtelomeric regions of different chromosomes.

The MLPA test also allowed us to simultaneously detect CNVs with all of the main kits used in this study (P064, P036 and P070); these changes were identified in ∼25.8% (8/31) of the patients.

One atypical duplication (in the *PRODH* gene) was only detected by the P356 kit, specific for chromosome 22, and one deletion in chromosome 8p23 (three probes) was detected with the P250 kit.

### ARRAY Analysis

The array technique was applied to all patients using different platforms (Agilent, Affymetrix or Illumina) and chip densities. The results showed that ∼14% (13/93) of the patients did not exhibit CNVs, while ∼86% (80/93) exhibited several different genomic alterations, including deletions, duplications and loss of heterozygosity (LOH). These changes were classified as pathogenic, benign or VOUS.

**Figure 3:**
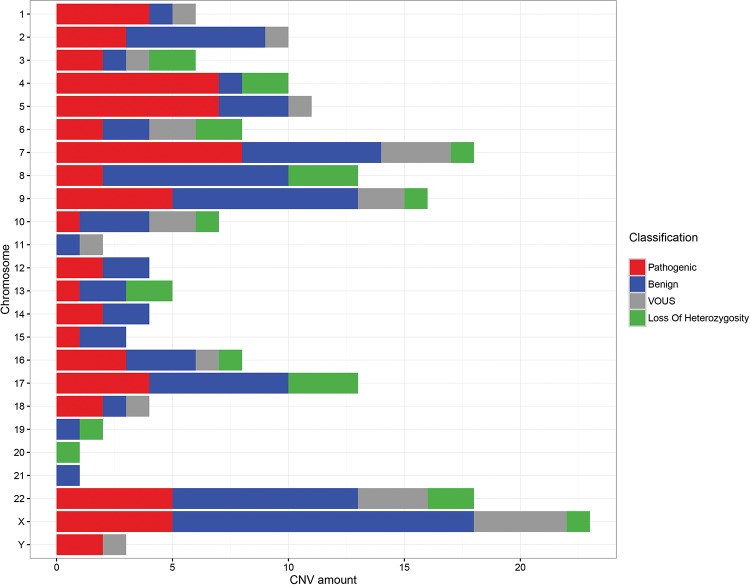
The number of CNVs identified on each chromosome via the array technique. The red bar indicates pathogenic CNVs; the blue bar indicates benign CNVs; the gray bar indicates VOUS; and the green bar indicates LOH.

Among the patients showing changes in the genome, we observed a 46.25% (37/80) detection rate for patients with benign and/or VOUS CNVs and a 53.75% (43/80) rate for patients with at least one pathogenic change (Figure 3).

Among the patients with pathogenic CNVs, ∼51.2% (22/43) exhibited only one alteration that was considered pathogenic, while ∼44.2% (19/43) showed at least two changes with important clinical significance, and ∼4.6% (2/43) of patients exhibited three or more pathogenic CNVs, possibly due to complex rearrangements. In several cases, these patients with pathogenic changes also displayed concomitant benign changes or VOUS.

Regarding the size of the changes, the majority of patients exhibited benign CNVs or VOUS ranging from 100 to 500 kb and pathogenic CNVs that were larger than 1 Mb.

## DISCUSSION

Establishing an unequivocal clinical and molecular diagnosis for patients with DD and MCA is essential for correlating genotypes and phenotypes and making genetic counseling more effective.

With advances in cytogenomic techniques, different syndromes can be better evaluated. Thus, for certain changes, specific genes are now highlighted as being responsible for most of the clinical features of a defined syndrome, whereas for others it is possible to determine alterations in an increasing number of critical regions associated with specific clinical characteristics 1,6.

Currently, the MLPA technique has become very useful for the detection of the main microdeletion/microduplication syndromes and subtelomeric imbalances, as it is a rapid technique that is able to detect typical changes correlated with specific phenotypes (e.g., Williams-Beuren syndrome or deletion of 22q11.2), in addition to being detecting small and/or atypical deletions and duplications in target regions 9,15,16. MLPA has the ability to assess more than 45 target regions in a single reaction without cell culture, making it a cost-effective and widely used technique for the validation of other methods, such as array-based analysis 12,15.

In this study, MLPA analysis using the P064 and/or P036 and P070 kits detected alterations in approximately 33.3% of patients. Using the same combination of MLPA kits, Jehee et al. 31 identified pathogenic changes in 21.8% of 261 patients with DD and MCA.

In a study performed on 258 patients with intellectual disabilities and dysmorphisms in 2007, the rate of the detection of alterations using several kits was 10.1%, among which only 5.8% were changes in regions correlated with syndromes, and 5.0% were associated with subtelomeric regions 15.

In the patients included in the present study, the changes identified with a specific kit for the main microdeletion/microduplication syndromes (P064) corresponded to ∼7.5% of all samples, or ∼22.6% of all changes, representing Williams-Beuren syndrome, duplications of chromosome 7q11 and deletions of chromosome 22q11.2. In addition, subtelomeric changes were found in ∼15.1% of the samples evaluated via MLPA, or ∼45.2% of the patients with copy number changes. In a similar study, the detection rate for alterations in the regions of the main microdeletion/microduplication syndromes was 6.6%, and the detection rate for subtelomeric alterations was 7.3% 10.

The percentage of copy number changes detected in the genome via MLPA depends on the criteria used to select patients, and the data obtained in this study corroborate the data reported in the literature for the regions corresponding to the main syndromes. However, the obtained values for subtelomeric regions were higher than those previously described by several authors.

A subtelomeric analysis conducted by Koolen et al. 14 detected changes in 6.7% of 210 patients with idiopathic intellectual disabilities. Two years later, Palomares et al. 32 detected alterations in 10% of patients with the same phenotypic characteristics using subtelomeric kits.

With the exception of two cases, all of the patients who presented only subtelomeric abnormalities exhibited two changes: one deletion associated with one duplication on different chromosomes, or two deletions or duplications. This set of changes in the same patients may result from complex rearrangements and translocations between chromosomes or regions of instability that are susceptible to rearrangements via DNA repair mechanisms.

We also detected changes with the three main kits used in this study (P064, P036 and P070) accounting for ∼25.8% of the CNVs identified among the abnormal results. These alterations may result from a microdeletion syndrome located near the telomere of a chromosome, such as 1p36 deletion syndrome, or complex rearrangements between different regions of chromosomes due to instability and microhomology.

In addition to the changes detected by the main kits used in this study, we were able to identify an atypical change involving a single gene (2 exons evaluated) using the P356 kit and a deletion in 8p23 (3 genes evaluated) using the P250 kit. These alterations are rare and difficult to detect because they involve specific genes or exons that are associated with few clinical characteristics, or a phenotype present in most patients, making it difficult to determine the correct kit to use.

An important limitation of MLPA is that the signal intensity of the probes varies according to DNA characteristics, including those associated with the extraction method, storage time, elution solution, degree of degradation (if present), and the presence of several types of contaminants, such as extraction reagents, proteins, RNAs, and salts. These influences can be minimized if all samples are prepared by the same technician using the same method. However, it is not always possible to eliminate this bias because samples may be sent from other locations, and storage times and DNA extraction methods may differ from the standard, which can cause artifacts during analysis that only a specialist can identify 8,18.

In our analyses using the MLPA technique, 4 patients showed inconclusive results with one or two of the kits, but none of these findings limited the detection of changes because the surveyed regions were represented in the other kits used in this study. These data highlight the importance of using different combinations of kits because one kit can act as a control for another, confirming the alterations detected and excluding false positive and negative results 10,32.

In a study performed by Marenne et al. 2, MLPA was used to validate data from arrays. DNA from 56 patients were analyzed via MLPA in two independent reactions, providing a concordance rate of 97.25%. Therefore, MLPA is a reproducible technique.

The sizes and breakpoints of chromosomal abnormalities can currently be determined with greater precision, accuracy and sensitivity using array techniques 6,19.

All of the patients included in our study were assessed using the array technique according to the availability of platforms or slides/chips in the laboratory (Agilent, Affymetrix or Illumina). The slides/chips differ in the technologies involved (CGH, oligonucleotides or beads) and in the number and spacing of probes distributed throughout the genome. Technologies with higher genome coverage provide more accurate breakpoint data and can be used to diagnose micro changes or several CNVs that were previously considered a single alteration (e.g., a normal region interposed by two affected regions). In these cases, the low coverage of several arrays may determine those changes to be a single deletion and not a complex rearrangement that may reflect a change in the patient’s phenotype 4,19,33.

A total of 93 samples were evaluated, and all of the different technologies employed proved to be satisfactory for detecting variations in the genome, which in most cases corroborated the clinical characteristics of each patient.

The data included results that were considered normal (without changes) for ∼14% of the patients. This rate is much lower than that described in the literature. In 2013, Vallespín et al. 27 evaluated 540 samples (patients with learning disabilities, autism and/or multiple congenital malformations) using a customized array with an average coverage of ∼43 kb and showed that no CNVs were detectable in 31.85% of the patients. In this study, the samples that were considered normal were assessed using Agilent 180K (2/13 patients), Agilent 60K (1/13 patients) and Illumina (10/13 patients) arrays, all of which exhibit a high rate of genome coverage. The results (particularly those from the Illumina platform; 65 samples), were considered normal because the majority of the evaluated patients had not received a suspected clinical diagnosis. These patients should be further evaluated and subjected to exome sequencing or targeted tests searching for mutations in specific genes or gene disruptions due to unbalanced translocations 4,20.

Among the patients who presented alterations in the genome, the array technique showed that 46.25% of the patients presented benign changes or changes of uncertain clinical significance, while 53.75% of the patients presented at least one pathogenic change.

Among the patients exhibiting alterations of clinical significance, the majority of patients presented only one or two pathogenic changes in the genome, which were or were not combined with other alterations, corresponding to ∼51.2% and ∼44.2% of the patients, respectively. Complex alterations with three or more pathogenic CNVs in different regions were observed in approximately 4.6% of the patients.

The detection rate of pathogenic alterations visualized in this study was much higher than the rates previously reported in several articles. Rosenberg et al. 34 investigated 81 patients with intellectual disabilities and facial dysmorphisms via the CGH array technique and concluded that 16% of the patients exhibited a pathogenic chromosomal imbalance related to their phenotype, while 4% of the patients exhibited changes of uncertain clinical significance. Gijsbers et al. 25 used several SNP array platforms to investigate patients with intellectual disabilities and multiple congenital abnormalities and detected alterations in 22.6% of 318 evaluated patients. Therefore, array analysis was considered the most appropriate test for the initial molecular investigation of patients with these characteristics and normal karyotypes.

Hochstenbach et al. 28 also recommended arrays as the first diagnostic test in this patient group. Based on analyzing many studies, they concluded that the rate of detection using arrays would correspond to at least 19% of pathogenic changes. Other studies have shown similar rates, regardless of the platform selected to diagnose patients with intellectual disabilities, malformations and/or neurological disorders and normal karyotypes 20,27,28.

Regarding the size of the observed changes, we identified the greatest number of patients with pathogenic CNVs that were larger than 1 Mb. These large changes usually involve more causative genes of a disease. However, the severity of the clinical manifestations in patients is not necessarily directly correlated with the size of the change but is correlated with location and gene content. Therefore, a small change can potentially reflect a more severe phenotype due to the pathogenicity of the altered gene 1,35.

With the implementation of SNP arrays, it has become possible to identify changes that were previously undiagnosed using CGH arrays. In this study, we identified four patients with LOH or UPD regions that can be correlated with recessive disorders 20,24,25.

The main challenge in analyzing the results of the arrays is determining which changes are significant for each patient, as it is common to identify more than one change per patient, and all of the changes could potentially influence the phenotype in many cases. The identification of benign and VOUS changes is associated with the increased array density used for diagnosis, as arrays with a greater number of probes are able to identify a greater number of microalterations and determine the breakpoints of these changes with higher accuracy. However, the identification of regions involving genes without an established function or regions that do not contain well-described genes will also increase 24,27,29.

All of the changes detected in the present study were checked against several international databases, including the DGV, Decipher and UCSC databases. Nevertheless, a more appropriate assessment of the changes identified in our patients would result in the creation of a database with information specifically from Brazilian people.

Most of the obtained results (∼97.8%) were concordant with each other for the regions investigated. However, not all of the results were in agreement, as the MLPA technique covers approximately 45 specific regions of the genome in each available kit, and this technique therefore depends on a clinical features and direction toward a specific target. Approximately 54.9% of the CNVs were not detected via MLPA compared with array analysis, and higher rates for this comparison (72-81%) are reported in the literature 2.

Despite the presence of the same alteration, one case was discordant in relation to the breakpoints detected via array analysis and the position of the MLPA probe. Therefore, to obtain a conclusive molecular diagnosis, other techniques should be applied to reevaluate the exact breakpoints involved.

All of the techniques employed in this study have advantages and disadvantages depending on the application and could potentially be applied together to obtain a complete molecular diagnosis.

Our findings showed that the interpretation of genotype-phenotype correlations in patients with complex genomic rearrangements is very difficult, but these results can directly contribute to the elucidation of new syndromes.

Arrays are a powerful tool for the identification and characterization of genomic abnormalities and can provide accurate diagnoses of previously unidentified or unexplained diseases that are suspected to have a genetic cause, contributing to appropriate clinical management of the affected patients. When an array is not available, MLPA with a combination of three kits (P064, P036 and P070) is a remarkable tool that can detect abnormalities in patients with DD and MCA 10,15,31.

Clinical and laboratory interactions with skilled technicians are required to target a patient for the most effective and beneficial molecular diagnosis, in which an appropriate clinical hypothesis is crucial for the successful detection of changes.

Patients exhibiting normal results or benign alterations may present a clinical phenotype due to balanced rearrangements with disruptions in several genes or mutations in specific genes. In this case, other molecular techniques are required to achieve a complete diagnosis, such as exome sequencing, which can detect changes in 80% of patients with developmental delays of unknown cause, and analysis using normal arrays 4,20.

## AUTHOR CONTRIBUTIONS

Zanardo EA wrote the paper and performed cytogenomic analysis. Dutra RL performed cytogenomic analysis and genotype-phenotype correlations. Piazzon FB performed the clinical evaluation and cytogenomic analysis. Dias AT, Novo-Filho GM and Montenegro MM performed molecular analysis and classical cytogenetic analysis; Nascimento AM prepared the samples and performed DNA extraction; Damasceno JG created the graphics and images. Madia FA and Costa TV discussed the results. Melaragno MI and Kim CA provided the samples and clinically assessed the patients; Kulikowski LD designed and coordinated the study. All authors read and approved the final manuscript.

## Figures and Tables

**Table 1:** Description of cytogenomic results obtained via the MLPA and array techniques.

ID	Array results				MLPA results					
	CNVs	Start - End	Size (pb)	Classification	Kit P064	Kit P036	Kit P070	Kit P250	Kit P356	Kit P029
01	del 17p11.2	17,626,111 - 17,640,000	13,889	Pathogenic	nml	nml	nml	-	-	-
02	del 19p13.12	14,729,069 - 14,768,462	39,393	Benign	nml	nml	nml	-	-	nml
03	del 22q11.21	21,034,808 - 21,572,202	537,394	VOUS	del 22q11.21 atypical (*SNAP29*)	nml	nml	del 22q11.21 atypical (*SNAP29* and *LZTR1*)	nml	-
04	dup 7q11.23→q22.1	74,480,670 - 99,700,362	25,219,692	Pathogenic	nml	nml	nml	-	-	-
05	dup 22q11.21	18,844,632 - 18,979,405	134,773	VOUS	nml	nml	nml	nml	inconclusive	-
06	dup 18q22.2→q22.3	68,090,674 - 68,756,043	665,369	VOUS	nml	nml	nml	nml	-	-
07	dup 17q21.31	44,204,373 - 44,788,310	583,937	Pathogenic	nml	nml	nml	nml	dup 22q11.21 atypical (*PRODH*)	-
	dup 22q11.21	18,877,787 - 19,008,108	130,321	VOUS						
08		No change			nml	nml	nml	-	-	-
09	dup Xq22.2	103,111,457 - 103,303,968	192,511	VOUS	nml	nml	nml	-	-	-
10	del 1q21.1→q21.2	146,516,199 - 147,828,939	1,312,740	Pathogenic	nml	nml	nml	-	-	-
11	dup 12q13.11	47,608,167 - 47,740,591	132,424	Benign	nml	nml	nml	-	-	-
12	del 8p23.2	4,814,896 - 5,044,296	229,400	Benign	nml	nml	nml	-	-	-
13	del 16p11.2	32,502,868 - 32,951,981	449,113	Benign	nml	nml	nml	-	-	-
14	del 6q25.2→q27	153,258,023 - 165,115,007	11,856,984	Pathogenic	nml	nml	nml	-	-	-
	dup Xp22.33	1,957,876 - 2,065,015	107,139	Benign						
15	dup 22q13.31	47,327,892 - 47,675,283	347,391	Benign	nml	nml	nml	inconclusive	nml	-
16	dup 22q11.22	22,314,463 - 22,580,314	265,851	Benign	nml	nml	nml	inconclusive	nml	-
17	del 22q11.21	18,877,787 - 21,462,353	2,584,566	Pathogenic	del 22q11.21 typical	nml	nml	del 22q11.21 typical	del 22q11.21 typical	-
	dup Xq28	152,667,088 - 153,878,001	1,210,913							
18		No change			nml	nml	nml	-	-	-
19		No change			dup 7q11.23 atypical (*FZD9*)	nml	nml	-	-	dup 7q11.23 atypical (*FZD9*)
20	del 6q24.3→q25.1	148,971,363 - 149,820,948	849,585	VOUS	nml	nml	nml	-	-	nml
21	del 17q23.3	61,947,000 - 61,977,500	30,500	Benign	nml	nml	nml	-	-	-
	del Xq22.1	99,904,100 - 99,905,800	1,700							
22	del 4q34.3→q35.2	179,962,284 - 190,790,881	10,828,597	Pathogenic	dup 5q35.3 typical	dup 5q35.3; del 4q35.2	dup 5q35.3; del 4q35.2	-	-	-
	dup 5q34→q35.3	160,148,716 - 180,712,253	20,563,537							
23	dup 12q24.32→q24.33	126,850,508 - 133,819,092	6,968,584	Pathogenic	dup 15q11.12 typical	dup 12q24.33; dup 15q11.2-cen	dup 12q24.33; dup 15q11.2-cen	-	-	-
	dup 15q11.1→q21.2	20,375,156 - 52,129,171	31,754,015							
24	del 9p24.3→p24.2	199,953 - 4,366,197	4,166,244	Pathogenic	nml	del 9p24.3; dup 18q23	del 9p24.3; dup 18q23	inconclusive	nml	-
	dup 18q12.3→q23	39,129,720 - 78,012,829	38,883,109							
25	dup 5p15.33→p13.3	37,692 - 33,434,546	33,396,854	Pathogenic	nml	dup 5p15.33; dup 14q11.2-cen	dup 5p15.33; dup 14q11.2-cen	-	-	-
	dup 14q11.2→q12	19,361,358 - 25,127,451	5,766,093							
26	dup 8p23.2	2,310,313 - 2,581,969	271,656	Benign	nml	nml	nml	nml	-	-
27	dup 9p13.1→p12	40,294,324 - 42,374,011	2,079,687	Pathogenic	nml	inconclusive	nml	nml	-	-
	dup 11q24.2	126,501,321 - 126,671,287	169,966	VOUS						
28	del 4p16.3→p16.1	48,283 - 6,471,246	6,423,143	Pathogenic	nml	del 4p16.3	del 4p16.3	nml	-	-
	dup 16p13.11	15,052,746 - 16,289,532	1,236,786							
	dup 10q11.22	46,947,635 - 47,741,321	793,686	VOUS						
29	del 16p12.2	21,599,125 - 21,740,231	141,106	Pathogenic	nml	nml	nml	-	-	-
30	del 7q11.23	72,722,981 - 74,138,121	1,415,140	Pathogenic	del 7q11.23 typical	nml	nml	-	-	-
	dup Xq24	117,394,974 - 117,742,647	347,673	VOUS						
	LOH 4q24-q26	102,641,428 - 118,463,264	15,821,836	4 regions – LOH						
	LOH 4q32.3-q34.1	166,848,001 - 175,764,593	8,916,592							
	LOH 17p13.2-p12	6,004,639 - 12,043,573	6,038,934							
	LOH 17q21.2-q22	38,640,744 - 54,902,055	16,261,311							
31	del 3p13→p12.1	74,143,047 - 85,618,308	11,475,261	Pathogenic	nml	nml	nml	-	-	-
	del Xp11.23	47,871,775 - 48,001,226	129,451	Benign						
	del 7p21.1→p15.3	20,703,948 - 21,582,516	878,568	VOUS						
32	del 22q13.2	41,036,329 - 41,640,297	603,968	Pathogenic	nml	nml	nml	nml	nml	-
33	del 8q24.23	137,730,280 - 137,850,011	119,731	Benign	nml	nml	nml	-	-	-
	dup Xp22.33	93,118 - 506,344	413,226							
34	del 8p23.2→p23.1	6,143,107 - 6,248,244	105,137	Benign	nml	nml	nml	-	-	-
	dup 10q11.21	45,212,898 - 45,359,483	146,585							
35	dup Xp22.31→p22.2	9,353,507 - 9,546,184	192,677	Benign	nml	nml	nml	-	-	-
	dup Xp22.2	11,047,140 - 11,608,207	561,067							
36	dup 15q26.3	100,351,154 - 100,589,056	237,902	Benign	nml	nml	nml	-	-	-
	LOH 3p22.1-p11.1	41,897,482 - 90,442,925	48,545,443	10 regions – LOH						
	LOH 3q11.1-q11.2	93,632,889 - 97,474,630	3,841,741							
	LOH 6q21-q25.1	107,328,319 - 149,605,182	42,276,863							
	LOH 6q25.3-q27	156,586,155 - 170,898,549	14,312,394							
	LOH 10q26.12-q26.3	122,697,234 - 131,869,597	9,172,363							
	LOH 13q32.1-q33.1	95,842,069 - 102,302,850	6,460,781							
	LOH 13q33.2-q34	106,386,553 - 115,106,996	8,720,443							
	LOH 16p13.13-p12.1	11,761,688 - 27,853,219	16,091,531							
	LOH 19p13.2-p13.11	8,386,306 - 16,372,158	7,985,852							
	LOH 20p12.2-p12.1	10,082,476 - 15,254,051	5,171,575							
37	del 4q32.1→q35.2	161,623,467 - 190,880,409	29,256,942	Pathogenic	nml	del 4q35.2	del 4q35.2	-	-	-
	dup 5p15.2	13,798,819 - 14,177,667	378,848							
38	del 2p11.2	90,027,810 - 90,247,720	219,910	Benign	nml	nml	nml	-	-	-
39	del 2q37.3	239,550,182 - 243,029,573	3,479,391	Pathogenic	dup 5q35.3 typical	del 2q37.3; dup 5q35.3	del 2q37.3; dup 5q35.3	-	-	-
	dup 5q35.1→q35.3	172,176,461 - 180,705,539	8,529,078							
40	dup 10q11.22	47,087,371 - 47,756,480	669,109	Pathogenic	nml	nml	nml	nml	nml	-
	dup 22q13.31	47,330,328 - 47,675,283	344,955	Benign						
41	del 9p23→p22.3	13,468,616 - 14,566,406	1,097,790	Benign	nml	nml	nml	nml	nml	-
42	del 7p22.3	45,130 - 1,691,646	1,646,516	Pathogenic	nml	del 7p22.3; dup 12q24.33	del 7p22.3; dup 12q24.33	-	-	-
	dup 12q24.22→q24.33	116,878,379 - 133,819,092	16,940,713							
43	dup 5p15.33	71,904 - 2,425,306	2,353,402	Pathogenic	nml	dup 5p15.33; del Yq12	dup 5p15.33; del Yq12	-	-	-
	del Yq11.221→q12	19,571,776 - 59,311,250	39,739,474							
44	dup 3q26.31→q29	174,466,591 - 197,845,254	23,378,663	Pathogenic	nml	dup 3q29; del 9p24.3	dup 3q29; del 9p24.3	-	-	-
	del 9p24.3→p23	204,104 - 11,659,355	11,455,251							
45	del 17p13.3	148,092 - 2,310,571	2,162,479	Pathogenic	del 17p13.3 atypical (*HIC* and *METTL16*)	del 17p13.3; dup 17q25.3	del 17p13.3; dup 17q25.3	-	-	-
	dup 17q25.1→q25.3	74,307,023 - 80,943,189	6,636,166							
46	del 2q33.1	203,291,000 - 203,312,000	21,000	Benign	nml	nml	nml	-	-	-
	del 3q28	189,360,000 - 189,364,000	4,000							
	LOH Xq21.1	78,667,293 - 82,400,000	3,732,707	1 region – LOH						
47	del 1q25.3	180,300,936 - 180,394,157	93,221	VOUS	nml	nml	nml	-	-	-
	dup 3q22.1	129,676,581 - 129,896,364	219,783							
	del 9p21.1	32,562,410 - 32,615,311	52,901							
48	dup 9p11.2	41,692,304 - 44,244,868	2,552,564	Pathogenic	nml	nml	nml	-	-	-
	del 9p11.2	44,727,846 - 44,824,251	96,405	Benign						
	dup 9p11.2	44,864,687 - 45,723,022	858,335	VOUS						
49	del 1p36.33→p36.32	564,620 - 2,456,203	1,891,583	Pathogenic	del 1p36 atypical (*TP73* nml)	del 1p36.33	del 1p36.33	-	-	-
	del 1p36.32	2,473,257 - 3,446,813	973,556							
	dup 1p36.32	3,474,630 - 3,641,681	167,051							
50	del 8q24.23	137,730,280 - 137,850,011	119,731	Benign	nml	nml	nml	-	-	-
	dup 7q11.22	71,021,037 - 71,272,257	251,220							
51	del 8q24.23	137,730,280 - 137,850,011	119,731	Benign	nml	nml	nml	-	-	-
	dup 14q11.2	20,213,937 - 20,379,392	165,455							
	LOH 7p15.1-p12.1	28,698,698 - 52,857,194	24,158,496	8 regions – LOH						
	LOH 8p23.1-p22	8,105,359 - 18,289,407	10,184,048							
	LOH 8q23.3-q24.23	114,783,837 - 137,679,805	22,895,968							
	LOH 8q24.23-q24.3	137,900,733 - 146,293,086	8,392,353							
	LOH 9q32-q34.11	115,745,240 - 130,633,433	14,888,193							
	LOH 17p13.3-13.1	53,011 - 9,193,945	9,140,934							
	LOH 22q12.3-q13.1	33,850,168 - 40,864,782	7,014,614							
	LOH 22q13.31-q13.33	45,136,360 - 51,169,045	6,032,685							
52	dup 4q28.3	131,880,992 - 132,305,574	424,582	Benign	nml	nml	nml	-	-	-
	del 22q11.23→q12.1	25,732,697 - 25,910,879	178,182							
	dup Xq22.2	103,179,170 - 103,303,968	124,798	VOUS						
53	del Xp22.13→p22.12	18,179,714 - 19,719,264	1,539,550	Pathogenic	nml	nml	nml	-	-	-
54	dup 14q32.33	106,067,618 -106,823,886	756,268	Pathogenic	nml	nml	nml	-	-	-
	dup 7q11.23	76,143,705 - 76,615,349	471,644	Benign						
	del 5q12.1	59,209,183 - 59,522,613	313,430	VOUS						
55	del 4q35.1→q35.2	185,821,036 - 190,880,409	5,059,373	Pathogenic	nml	del 4q35.2; dup Xq28	del 4q35.2; dup Xq28	-	-	-
	dup Xq27.1→q28	139,513,770 - 154,929,412	15,415,642							
	dup Xp22.33	2,139,005 - 2,319,653	180,648	Benign						
	dup Xq28	154,939,018 - 155,235,833	296,815							
56	dup 9p24→p23	46,587 - 13,014,232	12,967,645	Pathogenic	nml	dup 9p24.3; del 18q23	dup 9p24.3; del 18q23	nml	nml	-
	del 18q22→q23	70,657,389 - 78,014,582	7,357,193							
	dup Xp22.31	7,811,750 - 8,115,453	303,703	Benign						
57	del 2q37.3	239,550,182 - 243,029,573	3,479,391	Pathogenic	dup 5q35.3 typical	del 2q37.3; dup 5q35.3	del 2q37.3; dup 5q35.3	-	-	-
	dup 5q35.1→q35.3	172,246,068 - 180,705,539	8,459,471							
	dup 18q12.1	27,778,530 - 28,050,968	272,438	Benign						
58	del 4p16.3→p16.1	48,283 - 9,370,908	9,322,625	Pathogenic	nml	del 4p16.3; dup 8p23.3	del 4p16.3; dup 8p23.3	-	-	-
	dup 8p23.3→p23.1	176,818 - 6,974,050	6,797,232							
59	dup 4q26→q35.2	118,777,687 - 190,880,409	72,102,722	Pathogenic	nml	dup 4q35.2; del 7q36.3	dup 4q35.2; del 7q36.3	-	-	-
	dup 6q27	168,329,404 - 168,612,631	283,227	Benign						
	del 7p21.2	14,436,385 - 14,737,999	301,614	VOUS						
	del 7q36.3	158,498,994 - 159,119,486	620,492							
60	dup 6p22.3→p12.3	24,247,896 - 50,203,633	25,955,737	Pathogenic	nml	nml	nml	-	-	-
	dup 2q22.2→q22.3	143,387,612 - 145,082,658	1,695,046	VOUS						
	dup 10q11.22	46,972,140 - 47,681,957	709,817							
61	dup 2p25.3→p24.3	72,184 - 14,844,939	14,772,755	Pathogenic	nml	inconclusive	dup 2p25.3; del 4q35.2	del 4q35 (KLKB1)	-	-
	del 4q35.1→q35.2	186,468,992 - 190,880,409	4,411,417							
	dup 6q27	168,336,052 - 168,596,251	260,199	VOUS						
62	del 7q11.23	72,569,012 - 72,685,658	116,646	Pathogenic	del 7q11.23 atypical (*FZD9* nml)	dup Yp11.32; dup Yq12	dup Yp11.32; dup Yq12	-	-	del 7q11.23 atypical (*FKBP6*, *FZD9* and *TBL2* nml)
	del 7q11.23	73,082,174 - 74,267,872	1,185,698							
	del 7q11.23	74,298,092 - 74,601,104	303,012							
	dup Xp22.33	192,991 - 2,693,037	2,500,046							
	dup Yp11.31→q11.23	0 - 28,800,000	28,800,000							
	dup 7p14.3	33,134,410 - 33,193,210	58,805	Benign						
	del 13q31.3	94,422,000 - 94,480,000	58,000							
63	dup 16q24.1→q24.3	85,817,324 - 90,148,796	4,331,472	Pathogenic	nml	del 16p13.3; dup 16q24.3	del 16p13.3; dup 16q24.3	inconclusive	nml	-
	dup 14q11.2	20,213,937 - 20,425,051	211,114	Benign						
	del 16p13.3	105,320 - 203,254	97,934							
	dup 22q11.22	22,314,463 - 22,573,637	259,174							
	del 16p13.3	227,406 - 828,466	601,060	VOUS						
	dup Xq22.2	103,173,049 - 103,303,968	130,919							
64		No change			nml	nml	nml	-	-	-
65		No change			nml	inconclusive	nml	-	-	-
66	dup 10q11.22	47,084,916 - 47,741,321	656,405	Benign	inconclusive	inconclusive	nml	-	-	-
67	del 8p21.3→p21.2	23,148,930 - 23,310,904	161,974	Benign	nml	nml	nml	-	-	-
68		No change			nml	nml	nml	-	-	-
69	dup 10q11.21	45,212,898 - 45,359,483	146,585	Benign	nml	del 16p13.3	del 19p13.3	-	-	-
70	del Xp11.23	47,871,775 - 47,985,557	113,782	Benign	nml	nml	nml	-	-	-
71	dup 9p13.1→p12	40,294,324 - 42,374,011	2,079,687	Benign	nml	nml	nml	-	-	-
72		No change			nml	nml	nml	-	-	-
73		No change			nml	nml	nml	-	-	-
74	dup Xq28	152,667,088 - 153,903,395	1,236,307	Pathogenic	nml	nml	nml	-	-	-
75		No change			nml	nml	nml	nml	nml	-
76	dup Yq11.23	27,266,362 - 28,693,558	1,427,196	VOUS	nml	nml	nml	-	-	-
77		No change			nml	nml	nml	nml	nml	-
78		No change			nml	nml	nml	nml	nml	-
79	del 2p11.2	90,027,810 - 90,247,720	219,910	Benign	nml	nml	nml	-	-	-
	dup 21q11.2	14,687,571 - 15,214,708	527,137							
	del Xp21.3	27,151,611 - 27,337,941	186,330							
80	dup 2q13	110,863,908 - 110,982,530	118,622	Benign	nml	nml	nml	-	-	-
81	dup 7q21.3	95,467,621 - 96,178,713	711,092	Benign	nml	nml	nml	nml	nml	-
	del 9p23→p22.3	13,468,616 - 14,566,406	1,097,790							
	dup 22q11.23→q12.1	25,732,697 - 25,910,879	178,182							
82		No change			nml	nml	nml	-	-	-
83	del 8p23.1	6,982,980 - 12,483,094	5,500,114	Pathogenic	nml	nml	nml	del 8p23 typical	nml	-
	dup 2q22.3→q23.1	148,649,175 - 148,956,584	307,409	Benign						
	dup 5p13.2	36,902,936 - 37,159,877	256,941							
	dup 6p21.1	44,810,418 - 45,334,537	524,119							
	dup 8q22.2	100,111,153 - 100,528,645	417,492							
	dup 11p15.2	14,504,463 - 14,906,450	401,987							
	dup 13q31.3	92,492,127 - 92,815,210	323,083							
	dup 17q11.2	29,574,712 - 29,699,649	124,937							
84	dup 7q11.1→q11.21	61,074,194 - 62,403,985	1,329,791	Benign	nml	nml	nml	nml	nml	-
	dup 12p11.1	34,362,752 - 34,853,011	490,259							
	dup 17q11.2	29,444,844 - 29,562,294	117,450							
	dup 17q11.2	29,574,712 - 29,699,649	124,937							
85	del 13q12.12	23,548,470 - 24,960,000	1,411,530	Pathogenic	del 22q11 typical	nml	nml	del 22q11 typical	inconclusive	-
	del 22q11.21	18,886,915 - 21,463,730	2,576,815							
	dup 17q21.31	44,246,211 - 44,580,136	333,925	Benign						
86	del 22q11.21	18,889,490 - 20,312,668	1,423,178	Pathogenic	del 22q11 atypical	nml	nml	del 22q11 atypical	del 22q11 atypical	-
	dup 1p21.1	103,155,605 - 103,510,258	354,653	Benign						
	dup 5p13.2	36,816,661 - 37,158,123	341,462							
	dup 17q11.2	29,479,196 - 29,697,251	218,055							
87	del 22q11.21	18,886,915 - 20,312,668	1,425,753	Pathogenic	del 22q11 atypical	nml	nml	del 22q11 atypical	del 22q11 atypical	-
	del 15q11.2	24,357,212 - 24,472,002	114,790	Benign						
	del 16p12.2	21,578,388 - 21,839,340	260,952							
88	dup 7p22.3→p21.1	44,935 - 19,155,339	19,110,404	Pathogenic	dup 7p typical	dup 7p22.3	dup 7p22.3	-	-	-
	dup 7p21.1→p15.2	19,159,422 - 26,403,574	7,244,152							
	dup 2q24.3	166,821,406 - 166,939,893	118,487	Benign						
	dup 5p13.2	36,877,640 - 37,158,123	280,483							
	dup 9p24.1	5,112,844 - 5,252,074	139,230							
	dup 22q11.21	18,886,915 - 19,008,108	121,193							
89	dup 7q21.3	95,467,621 - 96,178,713	711,092	Benign	nml	nml	nml	-	-	-
	del 9p23→p22.3	13,468,616 - 14,566,406	1,097,790							
90	del 9p24.1	8,012,608 - 8,227,101	214,493	Benign	nml	nml	nml	-	-	-
	del Xq25	126,923,848 - 127,145,037	221,189							
	dup Xp22.33	1,921,638 - 2,065,015	143,377							
91	del 9p23→p22.3	13,466,329 - 14,566,406	1,100,077	Benign	nml	nml	nml	-	-	-
	dup 22q11.23→q12.1	25,650,648 - 25,910,879	260,231							
92		No change			nml	nml	nml	-	-	-
93	del 5q14.3→q15	90,124,906 - 94,954,205	4,829,299	Pathogenic	nml	nml	nml	-	-	-

Abbreviations: Nml, normal; dup, duplication; del, deletion; VOUS, variant of uncertain clinical significance; pb, base pairs.
